# Characterization of Lactic Acid Bacteria and Yeast from Grains as Starter Cultures for Gluten-Free Sourdough

**DOI:** 10.3390/foods12234367

**Published:** 2023-12-04

**Authors:** Seung-Hye Woo, Jiwoon Park, Jung Min Sung, Eun-Ji Choi, Yun-Sang Choi, Jong-Dae Park

**Affiliations:** 1 Research Group of Food Processing, Korea Food Research Institute, Wanju 55365, Republic of Korea; wooseunghye@kfri.re.kr (S.-H.W.); pjiwoon@kfri.re.kr (J.P.); jmsung@kfri.re.kr (J.M.S.); eunjichoi@kfri.re.kr (E.-J.C.); kcys0517@kfri.re.kr (Y.-S.C.); 2 Department of Biotechnology, College of Life Sciences and Biotechnology, Korea University, Seoul 02841, Republic of Korea

**Keywords:** gluten-free grains, lactic acid bacteria, yeast, starter culture, gluten-free sourdough bread

## Abstract

With the increasing number of people affected by gluten consumption-related diseases, adhering to a gluten-free (GF) diet is the most effective preventive measure. Herein, we aimed to isolate and characterize the functional properties of autochthonous lactic acid bacteria (LAB) and yeast from various GF sourdoughs to determine their suitability in starter cultures for sourdough preparation. Three LAB, *Weissella confusa* BAQ2, *Lactobacillus brevis* AQ2, *Leuconostoc citreum* YC2, and *Saccharomyces cerevisiae* BW1, were identified. The isolated LAB exhibited greater TTA, faster acidification rates, and higher acid tolerance than commercial LAB. *W. confusa* BAQ2 exhibited the highest EPS production, *W. confusa* BAQ2 and *L. brevis* AQ2 showed high maltose utilization, and *S. cerevisiae* BW1 exhibited the highest CO_2_ production rate. Accordingly, all four microbial strains were mixed for the starter culture. The sourdough prepared with starter cultures exhibited differences in gas production depending on fermentation time, which influenced the volume of GF bread dough. GF bread prepared with fermented sourdough exhibited a 16% higher specific volume and enhanced crumb firmness and elasticity than that prepared using non-fermented sourdough. Thus, autochthonous LAB strains isolated from various GF sourdoughs can be used together to improve the quality of sourdough bread, demonstrating their potential for use in starter cultures for GF sourdough production.

## 1. Introduction

Gluten, an insoluble protein comprising glutenins and gliadins, is the primary structuring agent in many baked goods in the bakery industry, contributing to the quality (texture and appearance) of the product [1]. However, the number of people affected by diseases related to gluten consumption, such as celiac disease, non-celiac gluten sensitivity, dermatitis herpetiformis, wheat allergies, gluten ataxia, and other chronic inflammatory diseases [2], has increased in recent years. Currently, strict adherence to a gluten-free (GF) diet is the most effective preventive measure against these diseases [3].

Baked goods such as breads, cakes, biscuits, pizza, and pasta are frequently made using wheat flour. Therefore, GF grain alternatives are being developed for individuals with gluten sensitivities. While GF grains can effectively increase nutritional value compared to conventional products, they have limitations in maintaining the morphological appearance of the product. Baking with GF grains results in inefficient gas expansion and retention owing to the lack of gluten networks, resulting in reduced bread volume, poor crumb properties, and poor flavor [4]. These factors hinder the manufacture of GF products. Current research has focused on the use of starch, protein, gum, hydrocolloids, and physical property improvers to overcome the limitations inherent in GF baking [5,6,7]. For instance, hydrocolloids improve bread texture, slow starch retrogradation, and increase water retention, thereby improving overall product quality [5]. Resistant starch can increase dough elasticity and improve crumb texture [7]. Transglutaminase promotes network formation by enhancing the baking potential of GF flour [8]. The use of chemical additives is at odds with consumer demand for natural products because of concerns regarding their health effects. In fact, consumers have recently shown increased interest in healthy and safe foods and, as a result, carefully evaluate the ingredients and sources when choosing a product [9]. Overall, a clean label indicates that a product is free of chemical additives, contains easy-to-understand ingredients, and is natural or has undergone limited processing [10]. Therefore, research is needed to replace additives, such as dough enhancers or preservatives, in bread manufacturing to produce healthier and higher-quality bread.

Baking with sourdough is effective in improving the volume and flavor of bread. During natural fermentation, the quality of the product depends on the microorganisms present in the dough. Sourdough fermentation using selected starter cultures can be an effective method for improving GF baking performance [11] because the quality of sourdough bread can be maintained by controlling microbiological stability and metabolites [12]. Sourdough is mainly fermented by lactic acid bacteria (LAB) and yeast, and their metabolic activity determines the development of taste and aroma, shelf-life extension, and nutritional and technical characteristics [13]. LAB used as a starter culture for sourdough can improve the processing quality of dough because of their acidifying properties, exopolysaccharides (EPSs), proteolytic activity, and metabolites produced during fermentation [13]. Yeast can coexist with LAB and exhibit reciprocal effects because of its ability to convert sugars quickly and efficiently to alcohol and CO_2_, its acid tolerance, and its ability to grow in the presence of acetic acid [14].

Most commercial starter cultures have been developed using wheat-based substrates, rendering them less efficient for GF sourdough production [4,15]. Strains commonly used in wheat fermentation, such as *Lactobacillus helveticus* and *Lactobacillus paracasei*, have shown difficulty dominating and competing with autochthonous strains, necessitating evaluation of starter robustness [16]. Considering the unique sugar composition of various GF flours, it is especially important to select strains with strong adaptability and competitiveness to establish a stable microbial community for GF sourdough. Several studies have been conducted to select suitable strains for the fermentation of GF substrates. For instance, *Weissella* spp. are more effective starter strains for maltose-poor, glucose-rich sorghum sourdough than the traditional sourdough strain *Fructilactobacillus sanfranciscensis*. Additionally, *Weissella* spp. efficiently produces glucooligosaccharides and EPSs [17]. Similarly, Moroni et al. [18] showed that *W. cibaria* is the dominant strain in buckwheat sourdough with the natural fermentation of GF flour. Natural fermentation of buckwheat and teff involves selected species that are not typically found in traditional sourdough, suggesting that GF flour can serve as a valuable source of new and competitive LAB and yeast species that can be used as starter cultures for GF sourdough production [18]. Together, the results of these studies show that the choice of microorganisms used in making GF sourdough can differ based on the type of sourdough. In this regard, studies are needed to isolate and characterize suitable autochthonous strains of microorganisms to use as starter cultures to exploit the potential of the GF flour matrix.

This study aimed to isolate autochthonous LAB and yeast from various GF sourdoughs and investigate their functional properties to determine their suitability as starter cultures for GF sourdough preparations. After characterizing the acidification ability, EPS production capacity, and acid resistance of the isolated strains, they were mixed and applied to buckwheat sourdough. The effect on final quality characteristics was evaluated using sourdough in bread production.

## 2. Materials and Methods

### 2.1. Isolation of LAB and Yeast from Fermented GF Dough

The dough was prepared by mixing 10 g of GF flour (corn, quinoa, or buckwheat) with 10 mL of water and allowed to naturally ferment at 30 °C for 24 h. Thereafter, naturally fermented 1 g of the dough was added to 9.0 mL of sterile saline, vortexed, and then serially diluted with the sterile saline solution. Appropriate 10-fold dilutions were spread on de Mann Rogosa Sharpe (MRS; Difco Laboratories, Detroit, MI, USA) agar and Potato Dextrose Agar (PDA; Difco Laboratories), respectively, and then incubated at 30 °C for 24 h. Distinct colonies were isolated randomly from the plates and purified by streaking on MRS agar and PDA media twice. This process was performed identically for all three types of dough (corn, quinoa, and buckwheat). Selected bacterial colonies were delivered to Macrogen (Seoul, Republic of Korea), and the bacterial species were identified using 16S or 18S rRNA sequencing followed by sequence alignment using the National Center for Biotechnology Information Database’s (NCBI) Basic Local Alignment Search Tool (BLAST). The 16S rRNA and 18S rRNA gene sequences were aligned with reference sequences exhibiting sequence homology from the NCBI database, utilizing the multiple sequence alignment program Molecular Evolutionary Genetics Analysis (MEGA), version 7.0. Phylogenetic analysis of the gene sequence data was performed using the neighbor-joining method, and distances were computed using the maximum composite likelihood method. The branching patterns were validated using the bootstrap program with 1000 bootstrap replicates. The BLAST algorithm was employed to retrieve homologous sequences in GenBank.

API 50 CH is used for the identification of lactic acid bacteria according to the manufacturer’s instructions (Biomerieux, Marcy-l’Etoile, France). Distilled water (10 mL) was dispensed into the incubation box, with the strip placed in the incubation box after the bacterial cultures had been introduced into the API 50 CHL medium (5 mL) with a concentration of 2 McFarland. Then, it was incubated at 30 °C for 24 h, after which the wells were filled with the bacterial suspensions by the line mark with the addition of mineral oil.

### 2.2. Characterization of LAB and Yeast Isolates

#### 2.2.1. Acidification

The acidification properties of the isolated bacteria were tested using skim milk. MRS medium (40 mL) containing 5% skim milk was inoculated with a 1% bacterial broth culture grown for 24 h and then incubated at 30 °C for 48 h. The total titratable acidity (TTA) produced by the lactic acid bacteria was determined by diluting 1 mL of the fermented bacterial broth in 9 mL of distilled water at 24-h intervals. The mixture was vortexed and expressed as the amount (mL) of 1 M NaOH required to achieve a final pH of 8.5. The TTA was determined using the Kramer and Twigg formula as follows:

$$Total\;titratable\;acidity\left(\frac{g}{100\;g}\right)=\frac{Volume\;of\;NaOH}{Volume\;of\;sample}\times 0.9$$

#### 2.2.2. Bacterial Growth in Varied pH Broths

We evaluated the ability of the isolated LAB and yeast to grow in the presence of acetic acid or lactic acid and tolerate low pH conditions. Isolated (*Lactobacillus brevis* AQ2, *Leuconostoc citreum* YC2, *Weissella confusa* BAQ2, and *Saccharomyces cerevisiae* BW1) and commercial bacteria (KCTC3102 *L. brevis*, KCTC3524 *L. citreum*, KCTC3499 *W. confusa*, and KCTC17612 *S. cerevisiae*) that had been stored at −70 °C were inoculated at 1% in 5 mL of MRS broth or PDB broth and pre-cultured at 30 °C for 24 h. The pre-cultured strains were then inoculated at 1% into modified MRS broth (pH 6.5, 5.5, 4.5, and 3.5) and PDB broth (pH 5.0, 4.5, 4.0, and 3.5) containing lactic acid and acetic acid, respectively, and incubated at 30 °C for 48 h. To analyze cell growth, samples were collected periodically, and the optical density at 600 nm was measured using a spectrophotometer (Jasco V-650 spectrophotometer, Jasco Co., Tokyo, Japan).

#### 2.2.3. EPS Production

The 24-h sub-culture was inoculated into 30 mL of MRS broth supplemented with 5% sucrose and incubated at 30 °C for 48 h. The bacterial culture was centrifuged for 10 min at 3560× *g,* and the supernatant was separated. The same volume of isopropyl alcohol was then added to the supernatant, and the mixture was refrigerated overnight. The precipitated EPS was recovered by centrifugation and dried to determine the yield by weight. The amount produced was expressed as mg/mL of broth medium.

The maltose fermentation abilities of the isolated (*L. brevis* AQ2, *L. citreum* YC2, and *W. confusa* BAQ2) and commercial (KCTC3102, *L. brevis*, KCTC3524 *L. citreum*, and KCTC3499 *W. confusa*) bacteria were determined using MRS broth containing 2% maltose. Each bacterial suspension was mixed with the modified medium and incubated at 30 °C for 24 h. Absorbance was measured over 24 h at 600 nm to prepare a growth curve.

#### 2.2.4. Gas Production Ability

The fermentation capacity was evaluated by measuring CO_2_ production. Isolated LAB (*L. brevis* AQ2, *L. citreum* YC2, and *W. confusa* BAQ2) and yeast (*S. cerevisiae* BW1) were activated in MRS and PDB broth, respectively, at 30 °C for 24 h. Subsequently, cells were collected by centrifugation, washed with distilled water, and finally resuspended in distilled water. The bacterial mixture (100 mL) was placed in a screw-cap bottle and incubated in a 30 °C water bath. CO_2_ production was measured using a WSF-2000MH-10W Fermograph III (Atto Co., Ltd., Tokyo, Japan) for 24 h. Values are expressed as milliliters of CO_2_ and the total volume of gas produced every 2 h.

### 2.3. Physicochemical Properties of GF Sourdough Bread

#### 2.3.1. GF Sourdough Bread Preparation

Before sourdough preparation, *L. brevis* AQ2, *L. citreum* YC2, *W. confusa* BAQ2, and *S. cerevisiae* BW1 were cultured at 30 °C for 24 h. The pellets from each culture were collected (3560× *g*, 15 min, 4 °C), washed with sterile water to remove residual media components, and resuspended in sterile water for sourdough preparation. The isolated LAB were mixed in a ratio of 1:1:1 to prepare 1 × 10^6–8^ CFU/mL. For sourdough production, 100 g of buckwheat flour, 65.8 g of water, 1.4 mL of LAB mixture (isolated three LAB), and 2.8 mL of yeast were mixed using a hand mixer (Braun, Kronberg, Germany) for 3 min. In the sourdough, the initial inoculum of LAB and yeast was approximately 1 × 10^2–4^ CFU/mL and 1 × 10^6–8^ CFU/mL, respectively. Subsequently, the sourdough was fermented at 30 °C for 0, 8, and 48 h and then freeze-dried. Thereafter, 45 g of freeze-dried buckwheat sourdough, 45 g of buckwheat flour, 74.7 mL of water, 1 g of salt, 1 g of sugar, and 0.66 g of commercial yeast were kneaded using a hand blender (Braun) for 3 min. The dough treated with freeze-dried GF sourdough was placed in a bread mold, fermented at 30 °C for 4 h, and baked in an oven at 165 °C for 30 min to prepare the GF sourdough bread.

#### 2.3.2. GF Dough Evaluation

The amount of gas produced in the GF bread dough prepared using GF sourdough that had been fermented for 0, 8, or 48 h was measured. Bread dough (30 g) was prepared by adding the sourdough as described in Section 2.3.1. Samples were placed in screw-capped bottles, which were then placed in a water bath maintained at 30 °C and connected to a WSF-2000MH-10W Fermograph III (Atto Co., Ltd.). The amount of gas produced was determined at 1-h intervals during incubation for 10 h.

The expansion ability of the bread dough containing sourdough was also measured. Bread dough (10 g) treated with sourdough that had been fermented for 0, 8, or 48 h was placed in a 50 mL graduated cylinder and incubated at 30 °C for 4 h, and then the dough volume was measured. The ability of bread dough to expand was calculated as the percentage increase in the initial dough volume.

#### 2.3.3. GF Bread Evaluation

Before measuring the specific volume of the GF sourdough bread, it was cooled at room temperature for at least 1 h. Loaf-specific volume (cm^3^/g) was analyzed using a Volscan Profiler (Stable Micro Systems, Godalming, UK) and calculated as the ratio of the volume (cm^3^) to the mass of bread (g).

The crumb texture was evaluated using a TA-XT2 texture analyzer (Stable Microsystems) equipped with “Texture Expert” software (6.1.16.0). Texture profile analysis of the bread crumbs was performed using a 35-mm diameter cylinder probe with a test speed of 0.50 mm/s. To determine crumb hardness, the bread was cut into 20-mm slices, and texture analyses were performed on the central slices. The crumb firmness was calculated from the force-distance curves obtained after two compression cycles.

The water content of the GF sourdough bread was determined according to AACC 44-15 guidelines in replicates.

The crumb color of the bread samples was measured using a Chroma meter CR-400 (Konica Minolta, Inc., Tokyo, Japan). Color and lightness values were expressed as L*, a*, and b*. Triplicate readings were obtained from different positions on the breadcrumbs, and the mean values were recorded.

### 2.4. Statistical Analysis

All experiments were conducted at least in triplicate, and the mean values were compared using analysis of variance (ANOVA). Subsequently, Duncan’s multiple range test was performed at a significance level of *p*  <  0.05. All statistical analyses were performed using SPSS software (version 25.0; SPSS Inc., Chicago, IL, USA).

## 3. Results and Discussion

### 3.1. Identification of Bacterial Isolates

**Figure 1:**
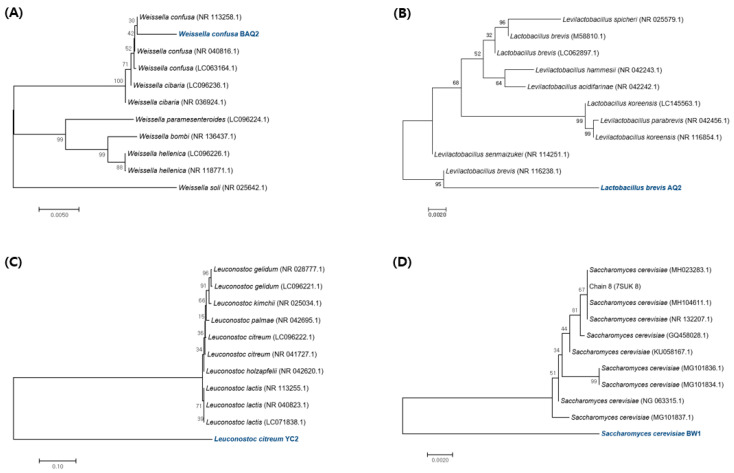
(**A**–**C**) Phylogenetic tree was constructed using 16S rDNA sequences compared to the most similar GenBank strains. (**D**) Phylogenetic tree was constructed using 18S rDNA sequences compared to the most similar strains in GenBank. The trees were constructed using the neighbor-joining method. The percentage of replicate trees in which the associated taxa clustered together in the bootstrap test (1000 replicates) is shown next to the branches. The tree is drawn to scale, with branch lengths in the same units as those of the evolutionary distances used to infer the phylogenetic tree. The evolutionary distances were computed using the Kimura 2-parameter method and are in units of the number of base substitutions per site. Blue font color indicated the isolated strains.

Among the strains isolated from various GF grains, lactic acid bacteria (LAB) were identified by 16S rRNA sequencing and yeast by 18S rRNA sequencing (Table 1). The bacterial species isolated from quinoa were *W. confusa* BAQ2 and *L. brevis* AQ2, whereas those from corn were *L. citreum* YC2. The strain isolated from buckwheat was identified as *S. cerevisiae* BW1 by 18S rRNA sequencing. To assess the phylogenetic relationships among the strains of pure-cultured LAB derived from gluten-free grains, their 16S rDNA gene fragments’ nucleotide sequences were determined. These sequences were then compared with those of the most closely related strains in the GenBank database to depict the resulting phylogenetic tree (Figure 1). Additionally, we analyzed the nucleotide sequences of the 18S rDNA gene fragments from isolated yeasts using the same methodology. This analysis aimed to investigate the phylogenetic relationships among the yeasts belonging to the *Saccharomyces* genus. The phylogenetic relationship of these isolated strains is displayed by a maximum composite likelihood-based neighbor-joining tree. In general, LAB isolated from sourdough or used as sourdough starters mostly belongs to the *Lactobacillus*, *Pediococcus*, *Leuconostoc*, and *Weissella* genera [19]. These strains play important roles when used as sourdough starters. The genus *Lactobacillus* produces aromatic compounds during sourdough fermentation, resulting in various types of flavors [20]. A mixed culture of *L. brevis* improves the organoleptic quality of GF bread by producing high concentrations of alcohol [4]. *Leuconostoc* and *Weissella* secrete dextransucrases that produce various types of dextran structures. A recent study showed that EPS produced by fermenting sorghum sourdough with *Weissella* improved the quality of GF products by replacing hydrocolloids [16]. Various metabolites (acetic acid, mannitol, and EPS) produced by *Leuconostoc* can create sourdoughs with processing properties that are suitable for GF bread production [21,22]. *S. cerevisiae* is a typical yeast species used in bread production and was isolated from buckwheat in this study. This strain maintains a symbiotic relationship in sourdough because of the proper oxidation-reduction balance between the metabolites produced by each strain when cultured with heterofermentative LAB [23]. Therefore, to further examine the properties of each strain, we explored whether the functional characteristics of the commercial strains (controls) could positively influence sourdough development when co-cultivated with the aforementioned isolates.

### 3.2. Functional Properties of Isolate Strains

#### 3.2.1. Acidification Capacity and Acid Tolerance

The strains isolated from the GF grains were evaluated for their suitability for producing GF sourdough. The evaluated factors included acidification properties, the ability to grow at low pH, EPS production, maltose solubility, and gas production ability. As a control, the abilities of the isolates were compared and analyzed using the same species purchased from the Korean Collection for Type Cultures (KCTC). The ability of LAB to acidify dough via chemical, metabolic, and enzymatic transformations is shown in Table 2. The changes in TTA can be attributed to differences in microbial metabolism and acidification rates among the strains. Further, these results indicate that the acidification rate of the isolated LAB was faster than that of the control LAB. Sourdough acidification can change the rheological properties of sourdough and affect enzymatic activity in cereals and bacteria because positive charges increase protein solubility [24]. In addition, the acidification ability of LAB is one of the factors that affects the quality, taste, and flavor of bread.

**Figure 2:**
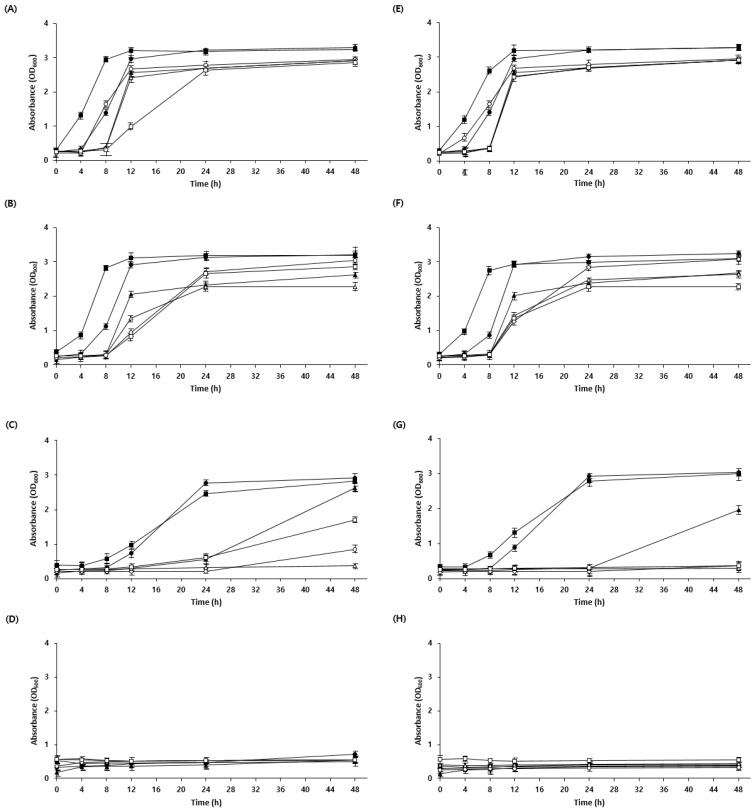
Growth curve of LAB cultivated at different pH values. (**A**–**D**) LAB were cultivated in an MRS medium using lactic acid to adjust the pH. (**A**) pH 6.5, (**B**) pH 5.5, (**C**) pH 4.5, and (**D**) pH 3.5. (**E**–**H**) LAB were cultivated in an MRS medium using acetic acid to adjust the pH. (**E**) pH 6.5, (**F**) pH 5.5, (**G**) pH 4.5, and (**H**) pH 3.5. Isolate *Lactobacillus brevis* AQ2 (●), isolate *Leuconostoc citreum* YC2 (▲), isolate *Weissella confusa* BAQ2 (■), KCTC3102 (○), KCTC3524 (△), and KCTC3499 (□) were grown for 48 h.

**Figure 3:**
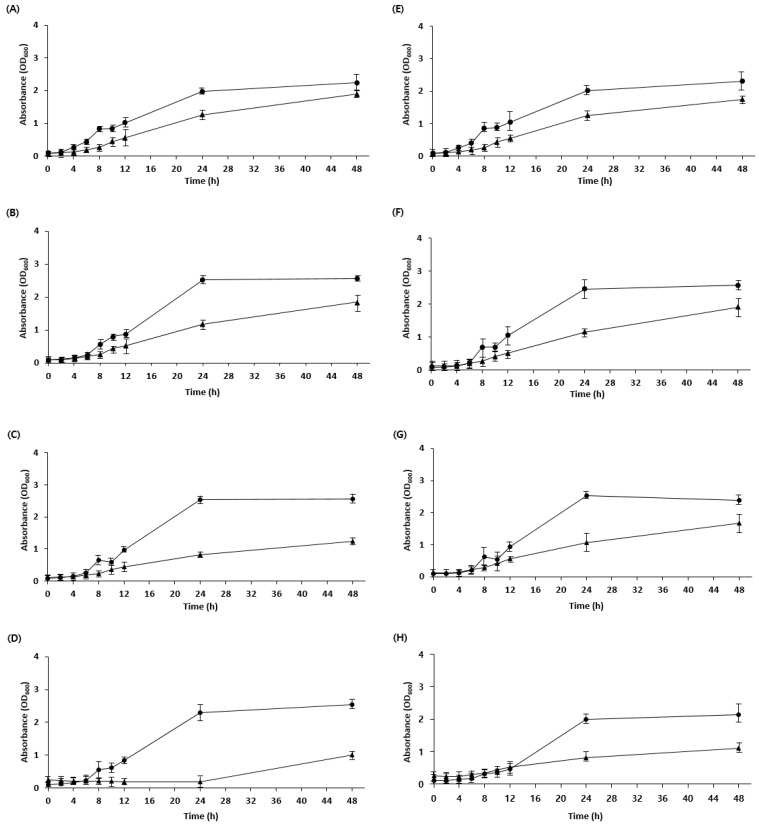
Growth curve of yeast strains cultivated at different pH values. (**A**–**D**) Yeast strains were cultivated in a PDB medium using lactic acid to adjust the pH. (**A**) pH 5.0, (**B**) pH 4.5, (**C**) pH 4.0, and (**D**) pH 3.5. (**E**–**H**) Yeast strains were cultivated in a PDB medium using acetic acid to adjust the pH. (**E**) pH 5.0, (**F**) pH 4.5, (**G**) pH 4.0, and (**H**) pH 3.5. Isolate *Saccharomyces cerevisiae* BW1 (●) and KCTC17612 (▲) were grown for 48 h.

Generally, the low pH of sourdough is due to the presence of acetic and lactic acids produced by LAB during fermentation. The low pH can inhibit the cellular uptake of nutrients, leading to energy depletion and reduced activity of metabolic enzymes [25]. Owing to evolutionary processes in microorganisms, some strains exhibit resistance to specific acids [26]. Therefore, acid tolerance was confirmed by adjusting the pH of the medium using lactic acid and acetic acid. *W. confusa* BAQ2, *L. citreum* YC2, and *L. brevis* AQ2 were tolerant to low pH adjustments using lactic and acetic acid, whereas KCTC3102 and KCTC3499 were intolerant, specifically to acetic acid, and KCTC3524 showed reduced acid tolerance overall (Figure 2). These results are consistent with those of a study showing that acid resistance is not necessarily bacterial species-specific but may vary among strains [27]. As acid stress is high in sourdough as a result of the low pH during fermentation, microorganisms require an acid resistance mechanism to maintain the stability of the sourdough ecosystem [28,29]. The isolated strains were expected to maintain a more stable ecosystem during sourdough fermentation because of their higher acid tolerance than that of the control group. Furthermore, the isolated yeast (*S. cerevisiae* BW1) showed high acid resistance at pH 3.5 compared with that of the control, indicating the possibility of the yeast and LAB coexisting in the sourdough (Figure 3). Overall, the strains required more time to adapt to low pH (below pH 4.5) than to high pH, which is consistent with observations from previous studies [30].

#### 3.2.2. EPS

The amount of EPS produced by the microorganisms was measured individually in all of the investigated groups (Table 2). The isolated bacterial strains *W. confusa* BAQ2 and *L. citreum* YC2 and the control bacterial strains KCTC3499 and KCTC3524 formed 30.51, 16.55, 24.35, and 15.74 mg/mL of EPS, respectively. This is consistent with research results showing that *Weissella* and *Leuconostoc* can produce EPS [31]. Although the isolated strains exhibited higher EPS production than the control strains, the differences were not significant (*p* < 0.05). Of all the measured strains, *W. confusa* BAQ had the highest EPS yield. In contrast, *L. brevis* AQ2 and KCTC 3102 showed relatively low EPS yields of 2.06 mg/mL and 1.98 mg/mL, respectively. These results are consistent with reports of significantly higher average yields of EPS among *Weissella* spp. than those of other LAB species [32]. The *Weissella* genus is one of the dominant strains in sourdoughs prepared from legumes and pseudocereals [33], and various forms of EPS can be produced using galactose, glucose, etc. [32]. EPS holds potential as a hydrocolloid replacement owing to its association with dough viscoelasticity and enhanced bread texture and volume [33]. Therefore, acidification and EPS production are the main metabolic activities of sourdough LAB that determine the functional properties of sourdough.

#### 3.2.3. Carbohydrate Utilization

To assess the ability of microorganisms to utilize carbohydrates, we used API 50 CHL, a standardized system consisting of 50 biochemical tests (Table 3). Regarding biochemical properties, *W. confusa* BAQ2, *L. citreum* YC2, and *L. brevis* AQ2 were generally positive for D-glucose, D-fructose, D-mannose, N-acetylglucosamine, amygdalin, esculin, cellobiose, and maltose. *L. citreum* YC2 was also specifically positive for mannitol, α-methyl-D-glucoside, and D-turanose. *L. brevis* AQ2 was positive for ribose, lactose, and 5-ceto-gluconate. In this study, sourdough was prepared using buckwheat flour with a sugar composition that included glucose, sucrose, maltose, and fructose [34]. As shown in Table 2, all of the isolated strains exhibited the ability to produce EPS, which aligns with that of strains typically associated with EPS production in traditional sourdough fermentation [19]. EPS can be produced from various sugars, including sucrose, glucose, mannose, fructose, and galactose [32]. When the isolated strain is used in buckwheat sourdough, it is expected to produce EPS using the sugars present in buckwheat flour. Species of *Weissella* use maltose to produce gluco-oligosaccharides, whereas species of *Leuconostoc* and *Lactobacillus* can commonly employ glucose or maltose as acceptor carbohydrates for glucan synthesis [16,19]. Isolated strains can produce different compounds depending on the carbohydrate types provided by the GF grains; further research is needed to investigate this.

**Figure 4:**
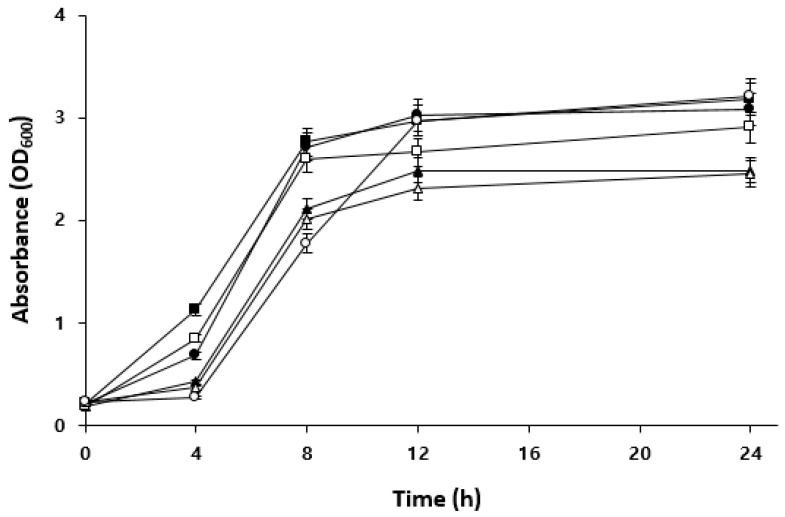
Comparison of maltose utilization among the isolated LAB. Isolate *Lactobacillus brevis* AQ2 (●), isolate *Leuconostoc citreum* YC2 (▲), isolate *Weissella confusa* BAQ2 (■), KCTC3102 (○), KCTC3524 (△), and KCTC3499 (□) were grown in MRS broth containing 2% maltose for 24 h.

Figure 4 shows the growth curves of each LAB strain in an MRS medium containing maltose. *W. confusa* BAQ2 and KCTC3499 showed higher initial absorbance values than the other strains, indicating high maltose utilization in the early stages of growth. Isolate *L. brevis* AQ2 and KCTC3102 exhibited low initial absorbance values but experienced rapid growth after 4 h of incubation, reaching absorbance values similar to those of *W. confusa* BAQ2 after 24 h. In particular, the isolate *L. brevis* AQ2 showed a similar absorbance value to that of *Weissella* even after 8 h of incubation, indicating a faster growth rate and greater environmental adaptability. *L. citreum* YC2, however, showed lower absorbance values than the other strains and relatively low maltose availability. These results indicate that the carbon source supporting the growth of *L. citreum* YC2 may differ from that of the other strains. Therefore, when the above strains are mixed, *L. citreum* YC2 may not compete with other strains for maltose, potentially contributing to the maintenance of their symbiotic relationship. In mixed cultures, the substrate utilization of each strain can impact the sourdough ecosystem. Carbohydrate metabolism is one of the most important metabolic pathways in sourdough. In particular, maltose is continuously produced along with glucose by the activity of amylase, an endogenous factor of sourdough, and can be used as substrates for microorganisms affecting the sourdough ecosystem [12]. Sourdough LAB affect the sourdough ecosystem by metabolizing maltose and releasing it as glucose for their consumption or that of other LAB and yeast [35]. Gobbetti et al. [36] reported that the lack of competition for maltose in a mixed culture of *Saccharomyces exiguus* M14 (maltose-negative) and *L. brevis* subsp. *lindneri* CB1 (syn. *L. sanfrancisco*) positively contributed to LAB metabolites in sourdough and bread flavor [36]. In general, *S. cerevisiae* can utilize various sugars, such as maltose, glucose, and sucrose, as carbohydrate sources [37]. Thus, the excretion of glucose by LAB can inhibit maltose utilization by competing yeasts such as *S. cerevisiae* [38]. As co-cultivation in a complex sourdough ecosystem results in interactions between microorganisms, it is important to examine individual carbohydrate utilization capacity and select the appropriate LAB–yeast combination.

#### 3.2.4. Gas Production

**Figure 5:**
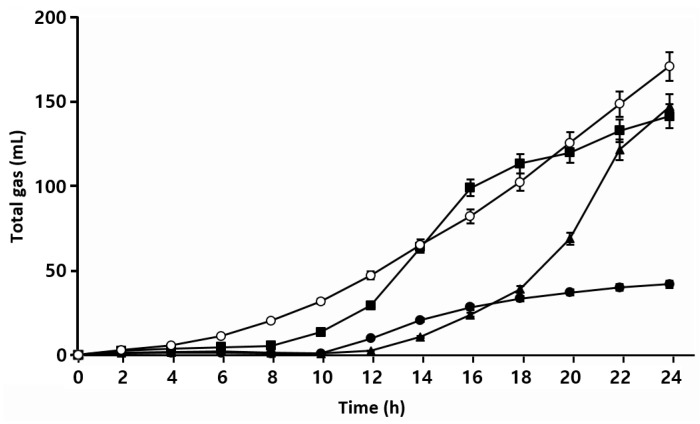
Total gas production of isolated strains was monitored using a Fermograph. Isolate *Lactobacillus brevis* AQ2 (●), isolate *Leuconostoc citreum* YC2 (▲), isolate *Weissella confusa* BAQ2 (■), and *Saccharomyces cerevisiae* BW1 (○) were grown at 30 °C for 24 h.

In sourdough bread production, fermentation gases are provided by yeast and lactic acid bacteria, which is one of the factors affecting the characteristics of sourdough bread [39,40]. Figure 5 shows that *S. cerevisiae* BW1 showed a steady increase in CO_2_ production after 2 h of incubation, with rates generally higher than those of the isolated LAB. *W. confusa* BAQ2 and *L. citreum* YC2 started producing CO_2_ after 8 and 12 h of incubation, respectively. *L. citreum* YC2 produced CO2 last among the strains but eventually showed a similar production to that of *W. confusa* BAQ2 at 24 h. *L. brevis* AQ2 exhibited the lowest CO_2_ production. Together, these results indicate that *S. cerevisiae* BW1 produced CO_2_ more rapidly and in larger quantities compared to that of the isolated LAB. Normally, *S. cerevisiae* converts sugars into CO_2_, energy, and biomass in the presence of oxygen and into ethanol, CO_2_, and glycerol in the absence of oxygen. This metabolic pathway starts as soon as *S. cerevisiae* is added to the dough and leads to the production of various secondary metabolites such as glycerol, organic acids, and flavor compounds [37]. The three isolated LAB commonly belong to heterofermentative LAB, and their most prominent metabolic activities in sourdough are acid and CO_2_ production [12]. However, the activity of each strain varies slightly depending on the LAB species and/or strain [41]. Our results revealed a difference in CO_2_ production rates among the four isolated microorganisms (heterofermentative LAB and yeast), and sourdough made from mixtures of these microorganisms is also expected to contribute to bread dough expansion.

### 3.3. GF Sourdough Bread Quality

#### 3.3.1. GF Dough Properties

The characterized strains (*W. confusa* BAQ2, *L. citreum* YC2, *L. brevis* AQ2, and *S. cerevisiae* BW1) were combined to establish the starter culture for gluten-free sourdough. Typically, the ecosystem within sourdough coexists with yeast and LAB [42,43]. Particularly, sourdoughs are characterized by a prominent diversity of lactic acid bacteria compared to yeast, suggesting that a substantial portion of the features attributed to sourdough are largely influenced by the metabolic outcomes of lactic acid bacteria [43]. Therefore, the composition of the starter culture used in sourdough fermentation is crucial in determining the quality of the bread. In most studies, a combination of two or more strains is employed [44,45,46,47]. Barley sourdough produced by selecting acid and salt-resistant lactic acid bacteria (*Lactobacillus plantarum* SAB15, *Lactobacillus brevis* SAB31) and yeast (*S. cerevisiae* SAM1–4) effectively enhances the quality of bread [44]. When various combinations of LAB and yeast strains isolated from traditional sourdough were combined, wheat sourdough produced with the combination of LAB *Lactobacillus brevis*, *Leuconostoc mesenteroides*, *Pediococcus acidilactici*, and yeast *S. cerevisiae*, *Kluyveromyces marxianus*, exhibited the most superior physicochemical and microbiological characteristics. Conversely, sourdough bread produced with a combination of *Lactobacillus plantarum*, *Lactobacillus brevis*, *Pichia kudriavzevii*, and *Wickerhamomyces anumalus* was evaluated as having the poorest quality [45]. Constructing an appropriate starter culture is a critical factor in enhancing the quality of sourdough. In this study, a preliminary experiment was conducted to select an effective combination of strains for the starter culture.

**Figure 6:**
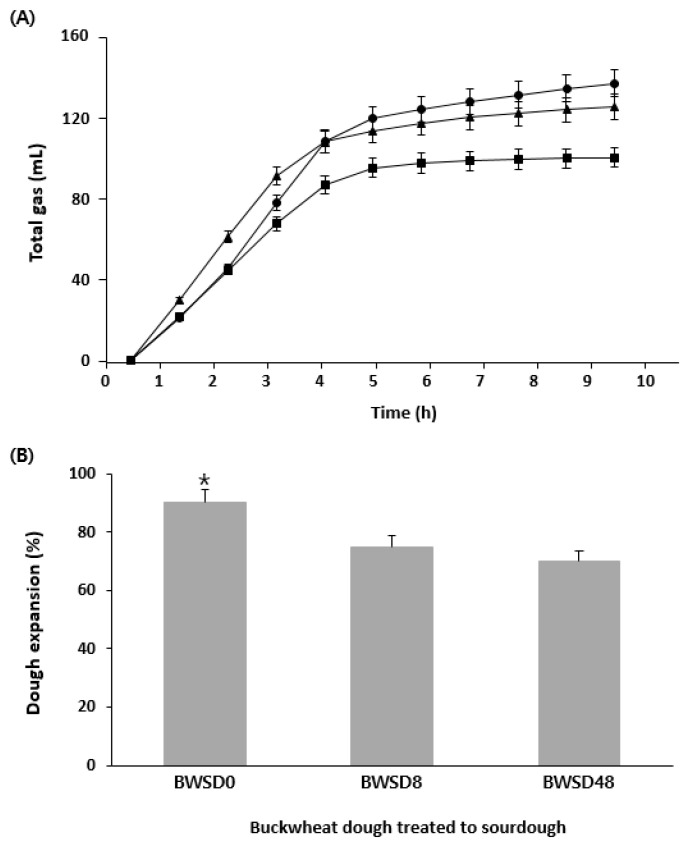
Comparison of GF bread dough containing GF sourdough prepared using different fermentation times. (**A**) Rheofermentographic profiles of the different doughs. Dough obtained by adding GF sourdough fermented 0 h (BWSD0) to the GF bread recipe (●); dough obtained by adding GF sourdough fermented 8 h (BWSD8) to the GF bread recipe (▲); dough obtained by adding GF sourdough fermented 48 h (BWSD48) to the GF bread recipe (■). (**B**) Degree of expansion of bread dough prepared with GF sourdough after 4 h fermentation. *: It showed the significant differences.

Three types of sourdough, fermented for 0, 8, and 48 h, were prepared, and the gases generated from bread dough containing each sourdough (BWSD0, BWSD8, and BWSD48, respectively) were analyzed. The bread dough also contained commercial yeast. Detailed recipes can be found in Section 2.3.1. The effect of GF sourdough prepared from the isolated strain combinations on CO_2_ production was analyzed using a Fermograph (Figure 6A). At the beginning of fermentation (before 4 h), BWSD8 produced the most gas at the fastest rate. However, BWSD48 produced the least gas, possibly due to high acid production over the long fermentation time, which may have inhibited commercial yeast growth, resulting in relatively low gas production. According to Liu et al. [48], low pH can prolong the lag phase of yeast and inhibit growth. BWSD0, with unfermented sourdough, produced the most gas due to the limited effect of LAB on the dough. The expansion of dough treated with sourdough was proportional to the gas production (Figure 6B). The dough expansion was significantly higher in BWSD0 (*p* < 0.05), which also exhibited the highest gas production. The Fermograph test was used not only to measure the amount of gas evolved but also to establish the fermentation time of the dough during the actual baking process. The Fermograph showed that the total amount of gas remained constant even after 4 h of fermentation, and no further gas was generated. Therefore, in this study, it was considered appropriate to set the fermentation time for the dough containing added sourdough to ≤4 h. Figure 6B presents the comparison of the dough volumes at this point. The volume of GF bread is one of several indicators of bread quality [49]. It is an important evaluation indicator because GF bread does not rise easily owing to the absence of gluten.

#### 3.3.2. GF Bread Properties

The quality characteristics of the sourdough GF bread are summarized in Table 4. The specific volume of bread after baking showed the opposite result to that of dough expansion. BWSD0, a GF bread with unfermented sourdough, exhibited characteristics similar to those of typical GF bread, as commercially available yeast dominated the dough during fermentation. In contrast, BWSD8 and BWSD48 demonstrated a 16% increase in specific volume compared with that of BWSD0. These results are consistent with previous studies showing that the addition of sourdough improved bread quality [13]. The sourdough fermented by the isolated LAB and yeast contains their metabolites; further, an appropriate amount of gas is effective in increasing the specific volume of bread [50]. When fermented dough is baked in the oven, it experiences gas expansion, leading to an increase in the volume of the bread (oven spring) [51]. However, in GF baking, since there is no gluten, gases are not retained, resulting in bread shrinkage [4]. However, in the case of bread containing fermented sourdough, such as BWSD8 and BWSD48, the ability to retain gas is attributed to substances produced in the starter culture [13]. EPS is recognized as a representative metabolite that can increase the viscosity of dough and impact the preservation of gas in bread [19]. Bread volume and crumb texture are the most intuitive indicators of bread structure, which can affect consumer acceptance. A basic bread recipe was used to rule out interference from other matrix components. In general, the addition of sourdough to GF bread produces an effect similar to creating a softer, textured crumb [50,52]. However, this study found that bread made with fermented sourdough (BWSD8 and BWSD48) was harder and more elastic than bread made with unfermented sourdough (BWSD0). Bread quality measurements revealed that BWSD0 exhibited low resistance to compression, resulting in it crumbling without returning to its original shape. In contrast, BWSD8 and BWSD48 exhibited higher resistance to compression and maintained their shapes after compression. Therefore, the increase in hardness and springiness in the texture profile shows that GF sourdough bread can maintain its original shape.

The characteristic color of buckwheat flour is related to polyphenol compounds [53,54]. Upon fermenting the buckwheat flour, the original color darkened, and the flour developed a slight red hue. The fermented sourdough tended to be darker and redder than the unfermented sourdough. Consequently, BWSD8 and BWSD48 became heavier and had increased yellowness over longer fermentation periods. Moreover, redness significantly increased in the BWSD48 group (*p* < 0.05). Because polyphenol compounds react sensitively to temperature and light, it is thought that buckwheat dough may change color if fermented for a long period of time. In a study by Fujita et al. [53], the color change in bread made with added buckwheat sourdough was consistent with our results. In addition, the overall favorability, including the color of the bread made using sourdough, was also highly evaluated [55]. Considering this, the change in bread color due to the addition of sourdough is expected to have a positive effect on consumer acceptance.

The addition of sourdough is known as a way to improve the quality of GF bread, and the efficacy of GF sourdough appears to be influenced the most by the characteristics of the fermenting microorganisms. Many studies have been conducted on the effects of various metabolites produced by starter cultures on bread quality. For the starter culture used in GF sourdough to maintain stability inside the dough, it is necessary to confirm the competitiveness and characteristics of the microorganisms that make up the starter culture against indigenous microorganisms. In this study, we isolated and characterized autochthonous strains from different GF sourdoughs and found that mixing these strains can enhance the quality of both buckwheat sourdough and bread made using sourdough. Therefore, this mixture is expected to exhibit stability in sourdoughs made from other GF flours. In addition to the numerous nutritional benefits of GF flours, the enhanced appearance (maintaining shape and changing color) of GF bread is also likely to have a positive impact on consumer acceptance. However, there are many factors that influence consumer acceptance. Due to the highly limited results of the presented bread properties, additional research is needed to explore potential influences, such as the flavor of bread formed by sourdough fermentation, beyond the bread’s appearance.

## 4. Conclusions

The starter culture, developed from autochthonous microorganisms isolated from GF sourdough, has considerably improved the volume and texture of GF sourdough bread, resulting in a resilient crumb. The starter culture consisted of three LAB and one yeast strain isolated from corn, quinoa, and buckwheat GF sourdoughs. The isolated yeast strain exhibited notable acid resistance, suggesting the potential for coexistence with LAB in sourdough. The LAB strains, isolated from various sources, displayed both high acidification ability and resistance to lactic and acetic acids, contributing to the stabilization of the sourdough ecosystem during fermentation. Moreover, their distinctive carbohydrate utilization capabilities are advantages for adapting to the unique sugar compositions in GF sourdough environments. GF bread made using a combination of these strains has increased volume, improved texture, and improved shape retention. These properties allow it to be used as a leavening agent, especially in bakery products, and have applications in a variety of products such as sponge cakes, cookies, and waffles. As a result, they hold the potential to serve as initiating starter cultures for the production of diverse GF sourdough breads. Further research is imperative to delve into potential influences, such as storage duration and flavor from the incorporation of fermented sourdough.

## Figures and Tables

**Table 1:** Identification and percent identity of lactic acid bacteria (LAB) strains isolated from gluten-free (GF) grains and identified using an API 50 CHL kit and 16S rRNA sequence analysis.

Source	Identification	API 50 CHL	16S rRNA
Quinoa	*Weissella confusa*	99.8%	99.0%
Corn	*Leuconostoc citreum*	99.8%	99.0%
Quinoa	*Lactobacillus brevis*	99.0%	99.0%

**Table 2:** Functional properties of LAB.

Bacteria	TTA (24 h)	TTA (48 h)	Exopolysaccharide (mg/mL)
*Weissella confusa* BAQ2	11.70 ± 0.90 ^a^	13.05 ± 0.90 ^a^	30.51 ± 8.57 ^a^
*Leuconostoc citreum* YC2	8.10 ± 0.00 ^c^	9.45 ± 0.45 ^c^	16.55 ± 0.64 ^b^
*Lactobacillus brevis* AQ2	10.35 ± 0.45 ^b^	10.8 ± 0.90 ^b^	2.06 ± 0.02 ^c^
KCTC3499 (*Weissella confusa*)	8.78 ± 0.23 ^c^	12.38 ± 0.23 ^a^	24.35 ± 0.86 ^a^
KCTC3524 (*Leuconostoc citreum*)	6.75 ± 0.45 ^d^	6.53 ± 0.23 ^d^	15.74 ± 0.07 ^b^
KCTC3102 (*Lactobacillus brevis*)	4.05 ± 0.45 ^e^	5.63 ± 0.68 ^d^	1.98 ± 0.69 ^c^

TTA, total titratable acidity. Data are presented as the mean ± standard deviation (n = 3). ^a–e^ Values in the same column with different letters are significantly different (*p* < 0.05).

**Table 3:** Carbohydrate utilization of the isolated LAB was determined using the API 50 CHL system.

Carbohydrate	Reaction			Carbohydrate	Reaction		
	*Weissella confusa* BAQ2	*Leuconostoc citreum* YC2	*Lactobacillus brevis* AQ2		*Weissella confusa* BAQ2	*Leuconostoc citreum* YC2	*Lactobacillus brevis* AQ2
Control	-	-	-	Esculine	+	+	+
Glycerol	-	-	-	Salicin	+	-	+
Erythritol	-	-	-	Cellobiose	+	+	+
D-Arabinose	-	-	-	Maltose	+	+	+
L-Arabinose	-	+	+	Lactose	-	-	+
Ribose	-	-	+	Mellibiose	-	-	-
D-xylose	+	-	+	Saccharose	-	+	+
L-xylose	-	-	-	Trehalose	-	+	+
Adonitol	-	-	-	Inulin	-	-	-
β-Methyl-xyloside	-	-	-	Melezitose	-	-	-
Galactose	+	-	+	D-Raffinose	-	-	-
D-Glucose	+	+	+	Amidon	-	-	-
D-Fructose	+	+	+	Glycogen	-	-	-
D-Mannose	+	+	+	Xylitol	-	-	-
L-Sorbose	-	-	-	β-Gentiobiose	+	-	+
Rhamnose	-	-	-	D-Turanose	-	+	-
Dulcitol	-	-	-	D-Lyxose	-	-	-
Inositol	-	-	-	D-Tagatose	-	+	+
Mannitol	-	+	-	D-Fucose	-	-	-
Sorbitol	-	-	-	L-Fucose	-	-	-
α-Methyl-D-Mannoside	-	-	-	D-Arabitol	-	-	-
α-Methyl-D-Glucoside	-	+	-	L-Arabitol	-	-	-
N-Acetyl glucosamine	+	+	+	Gluconate	-	-	-
Amygdalin	+	+	+	2-Ceto-gluconate	-	-	-
Arbutine	+	-	+	5-Ceto-gluconate	-	-	+

(+), positive reaction; (-), negative reaction.

**Table 4:** Characteristics of GF bread prepared with GF sourdough.

Bread Samples	Specific Volume (mL/g)	Moisture (%)	Texture Properties		Color Analysis		
			Hardness	Springiness	L*	a*	b*
BWSD0	1.044 ± 0.09 ^a^	48.61 ± 0.44 ^a^	1326.28 ± 210.75 ^b^	0.809 ± 0.00 ^b^	61.84 ± 0.26 ^a^	2.99 ± 0.06 ^b^	10.99 ± 0.05 ^c^
BWSD8	1.206 ± 0.08 ^a^	44.77 ± 0.29 ^c^	1905.55 ± 111.24 ^a^	0.903 ± 0.00 ^a^	59.52 ± 0.05 ^b^	2.84 ± 0.01 ^c^	11.28 ± 0.02 ^b^
BWSD48	1.212 ± 0.08 ^a^	45.82 ± 0.36 ^b^	1919.97 ± 149.70 ^a^	0.906 ± 0.03 ^a^	59.31 ± 0.09 ^b^	3.66 ± 0.04 ^a^	11.54 ± 0.08 ^a^

Data are presented as the mean ± standard deviation (n = 3). ^a–c^ Values in the same column with different letters are significantly different (*p* < 0.05). BWSD0, BWSD8, and BWSD48 represent bread dough containing sourdough fermented for 0, 8, or 48 h, respectively.

## Data Availability

Data are contained within the article.
